# Who knows best? A Q methodology study to explore perspectives of professional stakeholders and community participants on health in low-income communities

**DOI:** 10.1186/s12913-019-3884-9

**Published:** 2019-01-14

**Authors:** Neil McHugh, Rachel Baker, Olga Biosca, Fatma Ibrahim, Cam Donaldson

**Affiliations:** Yunus Centre for Social Business and Health, Glasgow Caledonian University, M201, 2nd Floor, George Moore Building, Cowcaddens Road, Glasgow, G4 OBA Scotland

**Keywords:** Health inequalities, Q methodology, Views, United Kingdom, Causes, Solutions

## Abstract

**Background:**

Health inequalities in the UK have proved to be stubborn, and health gaps between best and worst-off are widening. While there is growing understanding of how the main causes of poor health are perceived among different stakeholders, similar insight is lacking regarding what solutions should be prioritised. Furthermore, we do not know the relationship between perceived causes and solutions to health inequalities, whether there is agreement between professional stakeholders and people living in low-income communities or agreement within these groups.

**Methods:**

Q methodology was used to identify and describe the shared perspectives (‘subjectivities’) that exist on i) why health is worse in low-income communities (‘Causes’) and ii) the ways that health could be improved in these same communities (‘Solutions’).

Purposively selected individuals (*n* = 53) from low-income communities (*n* = 25) and professional stakeholder groups (*n* = 28) ranked ordered sets of statements – 34 ‘Causes’ and 39 ‘Solutions’ – onto quasi-normal shaped grids according to their point of view. Factor analysis was used to identify shared points of view. ‘Causes’ and ‘Solutions’ were analysed independently, before examining correlations between perspectives on causes and perspectives on solutions.

**Results:**

Analysis produced three factor solutions for both the ‘Causes’ and ‘Solutions’. Broadly summarised these accounts for ‘Causes’ are: i) ‘Unfair Society’, ii) ‘Dependent, workless and lazy’, iii) ‘Intergenerational hardships’ and for ‘Solutions’: i) ‘Empower communities’, ii) ‘Paternalism’, iii) ‘Redistribution’. No professionals *defined* (i.e. had a significant association with one factor only) the ‘Causes’ factor ‘Dependent, workless and lazy’ and the ‘Solutions’ factor ‘Paternalism’. No community participants *defined* the ‘Solutions’ factor ‘Redistribution’. The direction of correlations between the two sets of factor solutions – ‘Causes’ and ‘Solutions’ – appear to be intuitive, given the accounts identified.

**Conclusions:**

Despite the plurality of views there was broad agreement across accounts about issues relating to money. This is important as it points a way forward for tackling health inequalities, highlighting areas for policy and future research to focus on.

**Electronic supplementary material:**

The online version of this article (10.1186/s12913-019-3884-9) contains supplementary material, which is available to authorized users.

## Background

Health inequalities in the UK have proved to be stubborn, and health gaps between best and worst-off are widening [1]. While there is growing understanding of how the main causes of poor health are perceived among different stakeholders, similar insight is lacking regarding what solutions should be prioritised. Furthermore, we do not know the relationship between perceived causes and solutions to health inequalities, whether there is agreement between professional stakeholders and people living in areas with worse health or agreement within these different societal groups.

### Causes of health inequalities in the UK

A strong evidence base exists on UK socioeconomic inequalities in health [1, 2], described as “systematic differences in health between different socioeconomic groups within a society” [3]. There is also broad acceptance of the importance of socioeconomic disadvantage in explaining poorer health [1, 4]. Fewer studies have explored the general public’s understanding of health and causes of illness but there is growing interest in ‘lay knowledge’ and perceptions of causal attributions and acceptance of potential policy responses [5–8].

Although an earlier review by Blaxter [5] found individual behaviour rather than structural and environmental issues to be at the forefront of individuals’ conceptions of poor health, a recent meta-ethnographic review by Smith and Anderson [8] found that individuals had a more sophisticated understanding of how health is affected, with material and environmental factors gaining prominence. Importantly, studies undertaken in UK communities impacted by austerity suggest that economic and political structures, particularly neoliberalism, and psychosocial factors are seen as causes of poor health [9, 10] although behavioural and individualised factors are still viewed as partly to blame for health inequalities [10].

### Solutions to health inequalities in the UK

Despite an improved understanding of why health inequalities exist within the UK, this has yet to be translated into the implementation of transformative policies. While acting on ‘upstream’ social and economic determinants (causes of causes) is a primary concern, public health research, health policy and practice has been criticised for a ‘lifestyle drift’, focusing ‘downstream’, on modifying individuals’ health behaviours, such as smoking and drinking [11–13]. A number of reasons for this have been postulated. The evidence base for interventions further ‘downstream’ is easier to generate (the ‘inverse evidence law’), meaning this evidence base is more developed and easier for policymakers to draw upon [1, 14–16]. Political will to implement ‘upstream’ interventions is also often considered lacking as political systems prioritise short-term outcomes while ‘upstream’ outcomes are typically longer-term and, generally, require cross-sectoral action and a political mandate to implement radical policy solutions [17–20].

Failure to engage with the general public around solutions to health inequalities is a criticism levelled at researchers as top-down approaches have been prioritised over drawing upon and harnessing community voices [21]. While exploration of lay perceptions has been used to good effect in the area of causes [5, 8], similar work on potential solutions is lacking. The closest study to achieving this is an online survey of 502 UK and USA respondents that measured the relative importance of two social compared with nine behavioural factors related to health via a rank-ordering procedure [22]. The importance of social factors was vastly underestimated with respondents having a better sense of the relative importance of different behavioural risk factors. Elsewhere, qualitative studies in the USA [23] and Australia [24] found more emphasis was placed on individual behaviours and attitudes; Putland et al. [24] found this even among individuals who identified structural and social issues as the cause of health inequalities.

Amongst professional stakeholders in the UK, work exploring perceptions of solutions has produced conflicting results. MacKenzie et al. [25] elicited a range of different discourses across the social determinants of health using an innovative talk and draw technique with a small sample of health planners and practitioners in Scotland, the role of politics and power in shaping material disadvantage tended not to be explicitly recognised. Having asked health inequalities researchers to prioritise policies to address health inequalities, Smith and Eltanani [26] found consensus around economic proposals when prioritisation was based on respondents’ opinions while more support was found for lifestyle/behavioural interventions when prioritisation was based on available evidence. Neither of these studies explored relationships between respondents’ perceptions of causes and solutions.

### Exploring causes and solutions

While health inequalities research is very good at describing the problem, insight is lacking on perceived solutions to health inequalities and the relationship between views on causes and solutions. Such insight is needed to inform decisions around resource allocation, in terms of the development, piloting and evaluation of interventions and policies. Also it is not clear if different stakeholders, such as professionals and lay groups hold different views and thus if community voices are being represented in policy-making. Whilst professional stakeholders are likely to have well-rehearsed accounts in relation to their area of expertise, members of communities, who have not been asked such questions before, might respond better to more structured forms of data generation. In-depth interview techniques using open-ended questions and probes may mean some perspectives are not fully realised [27]. For example, in Putland et al.’s [24] qualitative study it was noted that in comparison to the number of reasons offered for health inequalities, solutions were more limited and narrowly focused. It is unclear whether these responses represented respondents’ views or if “the limited pallet of ideas” (24, p9) represented their struggle to readily articulate answers to open-ended questions. In this study we ask respondents to respond to a set of statements covering the range of reasons for health inequalities and ways to act upon them, using an approach that has potential to yield new insights.

The aim of this paper is to examine shared perspectives on, and relationships between, why health is worse in low-income communities (‘Causes’) and the ways that health could be improved in these same communities (‘Solutions’) among professional stakeholders and community participants. We introduce a new way of examining these issues – Q methodology.

## Methods

### Q methodology

Q methodology combines qualitative and quantitative techniques to study ‘subjectivity’ [28, 29]. Respondents rank order a set of statements of opinion onto a grid (card-sort). Factor analysis is used to identify patterns of similarity between different card-sorts (factors). Factors are represented by a distinctive ranking of the original statement set on idealised card-sorts; this represents how a respondent with a correlation coefficient of 1 with a particular factor would have rank-ordered the statements. These idealised card-sorts are used as the basis of interpretation from which a narrative of each factor is produced. An in-depth description of Q methodology is provided elsewhere (see – [29–31]).

Typically, Q studies only involve one card-sort. However, some previous studies have undertaken two card-sorts with the same sample [32–34]. In this study two card-sorts were designed to focus on i) why health is worse in low-income communities (‘Causes’) and ii) the ways that health could be improved in these same communities (‘Solutions’). Thus respondents consider a representation of the different opinions (statements) that exist on ‘Causes’ and then ‘Solutions’, in relation to their own point of view. The views of different types of respondents – professionals and community participants – can be compared using ‘factor loadings’ (correlation coefficients) representing the similarity between each respondents’ individual ranking of the statements and each factor.

### The statement set

The statement set is comprised of subjective views or opinions (not facts) on the topic in question. Any method can be used to access the conversational possibilities that exist on a given topic (‘concourse’), for example, interviews, newspaper articles or social media [35]. A representative sample of statements is then generated by removing repetition and overlap and being concerned with covering the range of issues that can be found [29].

In this study statements were drawn from surveys and interviews with low-income participants in a study of fair credit, health and well-being (FinWell – Chief Scientist Office CZH/4/1095), and self-complete response sheets at two research events. Questions relating to causes of, and solutions to, the worse health of low-income communities were included in the final questionnaires completed by low-income diary participants (*n* = 42) and during qualitative interviews with individuals from this same sample (*n* = 4). Then, at two research events (a seminar on financial diaries and health and a research showcase event at Glasgow Caledonian University), attendees (e.g. public health and social policy academics, policymakers, health and finance practitioners, Third Sector stakeholders) completed forms stating what they considered to be: the five main causes of ill health in low-income communities (*n* = 63 forms); and five ways to improve health in these same communities (*n* = 47 forms). In total we extracted 200 statements relating to ‘Causes’ and 120 statements relating to ‘Solutions’.

Statements were categorised using a well-known social determinants of health framework [36]; structuring the statement set in this way helps to examine coverage of the relevant issues. Duplicates were removed and similar statements merged, resulting in 32 ‘Causes’ and 36 ‘Solutions’ statements. These two sets were piloted with 12 individuals, including individuals from low-income communities and public health and social policy experts. Some statements were rephrased and some new statements added, on people having too many children (‘Causes’) and making more funding available for good primary health care, such as GP surgeries or community pharmacists, in these areas (‘Solutions’), with final statement sets of 34 ‘Causes’ and 39 ‘Solutions’. Statements were designed to form a complete sentence with a prefix, for example: “Health is worse in low-income communities because. .. of poor parenting” (see Tables 1 and 2).

**Table 1:** ‘Causes’ statement set and factor scores

#	Statement	Factors		
	Health is worse in low-income communities because. . .	F1	F2	F3
1	.. . people are unable to access space or places to meet others	0*	− 4*	*−* 3*
2	.. . people don’t have good support networks	0	*−*3*	0
3	.. . people feel like they are excluded from the rest of society	1	−3*	2
*4*	*.. . there isn’t enough community spirit*	*−2*	*−2*	*−2*
5	.. . of low levels of education	*−*1	−2	3*
*6*	*.*. *. of unpredictable finances*	*3*	*2*	*2*
7	.. . there is a lack of insight into what these communities need	1*	0*	− 2*
8	.. . people see others in society with status symbols like expensive phones or cars which make them feel bad about their own situation because they can’t afford them	*−*1	*−*1	−2
9	.. . of the stress of making hard decisions like “do we eat?” or “do we heat?”	4	4	0*
10	*.*. . people don’t get to experience the outdoors like being in the mountains, forests or by the sea	−1*	− 2	− 2
11	.. . there is a lack of good quality, affordable housing	2	1	3
12	.. . there aren’t things for young people to do in their community	0	−1	−1
13	.. . of how the welfare system works	4*	1	2
14	.. . people struggle to get access to services that are available	2*	− 4*	0*
*15*	*.*. *. many people don’t have jobs that are secure, meaningful or that give them a sense of purpose*	*3*	*3*	*3*
16	.. . people feel a sense of hopelessness from not being in control	1	1	4*
17	.. . people lack the ability to look after themselves	*−*3*	−1	0
18	.. . people can struggle with complicated family life, sexual, emotional or physical abuse	1*	*−* 1*	4*
19	.. . the culture of the community means people don’t have ambitions or goals	*−* 2*	1	1
20	.. . people are labelled, stereotyped and talked down to, they are not treated as individuals	2*	1	1
21	.. . the views of these communities aren’t taken into account	1*	*−* 2	−1
22	.. . it is difficult to leave an area to start a new life	0	−3*	0
23	.. . the people in these communities can’t cope with unexpected events or costs	0	3	1
*24*	*.*. *. these communities tend to be dirty, polluted or in poor condition*	*−1*	*−1*	*−1*
25	.. . people don’t have a way to travel, they can’t afford a car or public transport	0	0	−3*
26	.. . having less money increases the cost of things people need like electricity or loans	3	2	1
27	.. . of poor parenting	−3*	0*	2*
28	.. . people in these communities don’t follow health advice	−2*	2*	− 1*
29	.. . people don’t feel safe where they are living	−1	0	1
30	.. . there is a culture of dependency and laziness in these communities	−4	4*	− 4
31	.. . people in these communities don’t take responsibility for their own health	−3*	0	0
32	.. . governments don’t invest in these communities	2	2	−3*
33	.. . people have too many children	−4	0*	− 4
34	.. . people focus on short-term pleasures rather than thinking about the future	− 2*	3*	− 1*

*Indicates distinguishing statements. Italics indicate consensus statements

**Table 2:** ‘Solutions’ statement set and factor scores

#	Statement	Factors		
	Health could be improved in low-income communities by. . .	F1	F2	F3
1	.. . making free childcare available and accessible	2	−2*	3
2	.. . spending more on the NHS	−1	2	2
3	.. . providing better support to rehabilitate prisoners, ex-offenders or people who have had addiction problems	2	−1	0
4	*.*. . supporting industries, companies or sectors that can provide ‘good work’	1	2	4*
5	.. . investing in community activities and groups which give people something to do	5*	1*	*−* 1*
*6*	*.*. *. focusing on how we better support vulnerable individuals like young men, young mums or older people*	*3*	*2*	*1*
7	.. . increasing the availability of, and access to, social care services in these areas	0	0	3*
8	.. . helping people to develop their strengths	4	2	−2*
9	.. . helping people to make relationships with others so that they have someone to look out for them or to turn to when things get hard	4*	0	−1
10	*.*. . making it possible for people to access affordable, flexible loans when they need them	−1	−3*	1
11	.. . increasing the tax on things that are bad for people like alcohol, sugary food and drink or fatty foods	−3	−2	2*
12	.. . improving the quality of housing for people on low incomes	0	2	4
13	.. . making sure that people have enough money each month to pay for their basic needs like rent, food, clothing, heat for their home	2*	5	5
*14*	*.*. *. cutting welfare benefits*	*−4*	*− 5*	*−5*
15	.. . making sure that everyone who wants a job can get a job	1	1	4*
16	.. . legalising drugs	−3*	− 5*	− 1*
17	.. . making sure that everyone in society has similar opportunities	0	*−*2*	1
18	.. . by raising the taxes that people pay in a fair way	−2*	*−* 4*	5*
19	.. . providing ways for people to talk about and deal with mental health issues	2	4*	0
20	.. . better educating children about health from a young age	3*	5*	− 2*
21	.. . making sure communities have a say in any decisions that will affect them	5*	0	2
22	.. . providing services that help people to organise their money like financial advice	0	4*	− 1
23	.. . providing safe ways for individuals to own their home, a car, things like that without getting into debt that they can’t repay	−2	−1	1*
24	.. . encouraging children to have goals and to have the confidence to meet them	3	3	0*
25	.. . having more health campaigns	−3	0	−3
26	.. . people taking responsibility for themselves	−2*	1*	*−* 4*
27	.. . finding more ways for people from different groups or different communities in society to mix together	1*	− 2	−2
28	.. . improving the availability and price of public transport	−1	−1	1
29	.. . helping communities to own land, buildings or other assets in their community	−1	− 3*	− 1
*30*	*.*. *. reducing the price of things that are good for you like healthy food*	*1*	*3*	*2*
31	.. . providing coaching sessions for good parenting	−2	−1	−3*
32	.. . denying healthcare to people who are responsible for their own condition like smokers or fat people	−5	−4	−4
33	.. . stopping benefit payments to those spending their money on things that are bad for their health	−5	1*	− 4
34	.. . why should we do anything? If people want to make bad choices for their health then let them	−4	−1*	− 5
*35*	*.*. *. improving the environment of the community so that it is easier for people to be active outside*	*−1*	*0*	*0*
36	.. . by controlling what shops in these communities can sell	−4	−4	−3
37	.. . making more funding available for good primary health care, such as GP surgeries or community pharmacists, in these areas	0*	3	3
38	.. . these communities deciding what needs to be done to improve health and then doing it	4*	− 3	−2
39	.. . preventing payday or doorstep lenders from taking advantage of vulnerable individuals	1	4*	0

*Indicates distinguishing statements. Italics indicate consensus statements

### Data collection

As in most Q studies respondents were purposively selected to identify those with distinct, strong and different views rather than to represent a population [37]. There is no set sample size in Q studies. Instead concepts of data saturation are used, with sampling closing when a stable set of factors are identified and new card-sorts only confirm existing factors.

We selected from two groups: professional stakeholders and community participants (see Table 3). For the former, individuals with a variety of professional expertise were targeted, including, health care professionals, community development workers, public health experts, social policy and public health academics, financial services practitioners, policymakers, social activists and charity workers. For the latter group, participants from the FinWell study and individuals living in low-income communities were targeted. Non-FinWell community participants with a low-income and who claimed welfare benefits were identified through a market research company.

**Table 3:** Summary characteristics of full respondent sample (*n* = 53) and respondents defining the factors

				‘Causes’ – Defining* Sorts			‘Solutions’ – Defining* Sorts		
		N	%	C-1 (*n* = 27)	C-2 (*n* = 5)	C-3 (*n* = 6)	S-1 (*n* = 15)	S-2 (*n* = 10)	S-3 (*n* = 9)
Age	18–30	6	11%	3	1	0	1	1	2
	31–50	31	58%	15	2	3	9	5	5
	51–64	14	26%	7	2	3	4	4	1
	65+	2	4%	2	0	0	1	0	1
Gender	Female	30	57%	15	2	4	6	6	7
	Male	23	43%	12	3	2	9	4	2
Expertise/Experience	Academic	5	9%	2	0	0	0	0	3
	Community Development	7	13%	5	0	2	4	0	0
	Health/Social Care	6	11%	3	0	2	1	0	3
	Policy	7	13%	5	0	1	2	0	2
	Financial Services	3	6%	3	0	0	1	0	1
	Diarist - Business	9	17%	4	1	0	4	3	0
	Diarist - Personal	4	8%	1	2	1	0	2	0
	Diarist - Money Advice	5	9%	1	1	0	0	4	0
	Diarist - Control	3	6%	1	1	0	1	1	0
	Focus Group – Low-income Community	4	8%	2	0	0	2	0	0

*Defining card-sorts have a significant association (*p* < 0.01) and are only significant on one factor

**Fig. 1:**
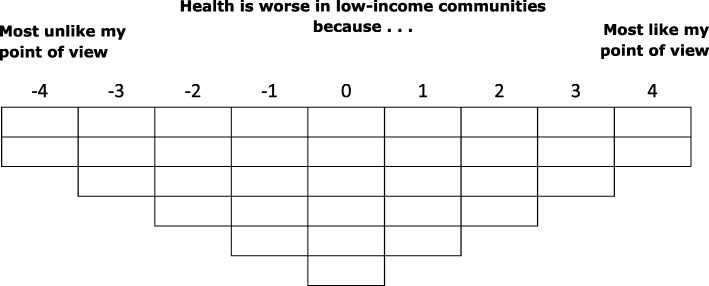
Causes’ Sorting-grid

**Fig. 2:**
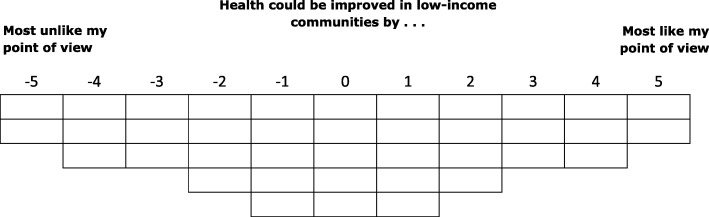
Solutions’ Sorting-grid

Each respondent completed two card-sorts (‘Causes’ and ‘Solutions’) administered by a member of the research team or a trained research assistant. A standardised introduction set the context (see Additional file 1). Respondents were presented with a shuffled set of statement cards at the beginning of each card-sort (see Tables 1 and 2) then asked to consider each statement in turn before placing it in one of three piles: ‘like my point of view’, ‘neutral’ or ‘unlike my point of view’ (the condition of instruction). Respondents were then guided through a rank ordering of statements onto a grid reflecting the number of statements in each set (see Figs. 1 and 2). Sorting grids were quasi-normal shaped: ‘Causes’ ranged from − 4 (most unlike my point of view) to + 4 (most like my point of view) and ‘Solutions’ from − 5 (most unlike my point of view) to + 5 (most like my point of view). Statements were placed column by column working towards the centre of the grid.

Following each card-sort a post-sort interview was conducted during which respondents were asked open-ended questions with follow-up probes about their card-sort. First, respondents were asked to sum-up their views and make general comments on why health is worse (‘Causes’), and how health could be improved (‘Solutions’), in low-income communities. More specific questions were then asked about statements placed at the extreme end of their grids. This short interview was audio-recorded and transcribed verbatim and the qualitative data were used to aid the selection of the factor solution and the interpretation of factors.

### Analysis

Each dataset – ‘Causes’ and ‘Solutions’ – was analysed separately in the first instance using PQMethod [38]. Following Watts and Stenner [29], centroid factor analysis was followed by Varimax rotation to identify a small number of shared viewpoints (factors) based on the correlations between respondents’ card-sorts. The selection of a factor solution was based on quantitative and qualitative criteria. Convention in Q factor analysis generally places greater emphasis on the interpretability of factors in relation to the qualitative accounts of defining card-sorters, and in relation to theory, than on the statistical tests to determine the best factor solution. Thus the factor analysis reported here was centred on the interpretation of factors, whilst examining some statistical features.

As the purpose is to identify shared viewpoints, we required that at least two card-sorts should *define* each factor [39]; defining card-sorts have a significant association with a factor and are only significantly associated with one factor [29]. The percentage of explained variance – or shared meaning – accounted for by any factor solution is also a relevant consideration, with an acceptable level of explained variance generally considered to be 35–40% or above [40].

However, it is not enough for a factor solution to be statistically acceptable (it is also possible for more than one solution to exist), factors must be interpretable with coherent and comprehensible narratives. This is assessed in part by the extent to which the accounts represented by the idealised card-sorts are consistent with the views expressed in the post-sort interviews by those whose card-sorts define each factor. Defining card-sorts are used to produce an idealised card-sort for each factor, based on a weighted averaging, such that defining cards-sorts with a higher association receive a greater weighting in the production of the idealised card-sort [29, 39].

Interpretation focusses closely on these idealised card-sorts and the relative placement of all statements for each factor. Particular attention is given to *characterising*, *distinguishing* and *consensus* statements in refining interpretations. Characterising statements are those placed near the poles of the sorting grid, for example in positions − 5, − 4, + 4 and + 5 of a grid shaped from − 5 to + 5. Distinguishing statements are positioned significantly differently for one factor compared to the same statement in another factor and consensus statements are not statistically significantly different between any pair of factors. For each factor, qualitative data from defining card-sorts were also used to understand respondents’ views, the rationales and reasons behind their card-sorts and their interpretation of the statements. This helped to enrich factor interpretation and in the factor descriptions particular quotes were selected to explain a point or illustrate, in respondents’ own words, their point of view (see Additional file 2).

Relationships between ‘Causes’ and ‘Solutions’ were explored qualitatively by examining factor descriptions, and quantitatively by examining correlations. In order to examine the relationship between respondents’ views on ‘Causes’ and ‘Solutions’, the factor loadings (see Table 4) of each ‘Causes’ factor were correlated with the factor loadings of each ‘Solutions’ factor. This indicates, for example, whether respondents who correspond to ‘Causes’ Factor 1 (C-1) also correspond to ‘Solutions’ Factor 1 (S-1).

**Table 4:** Respondents’ expertise, experience and factor loadings*

		Factor loadings - Causes			Factor loadings - Solutions		
ID	Expertise/ background	C-1	C-2	C-3	S-1	S-2	S-3
PS13	Academic Social Policy Researcher	**0.87X**	−0.19	0.09	0.17	0.26	**0.86X**
PS16	Financial Services Practitioner	**0.81X**	0.16	0.30	**0.47**	0.09	**0.66**
PS04	Academic Health Policy	**0.80X**	0.00	0.34	0.32	0.09	**0.81X**
PS01	Public Health Consultant	**0.80X**	−0.02	0.21	0.13	− 0.12	**0.89X**
PS21	Community Development Officer	**0.78X**	−0.10	0.34	**0.55**	0.19	**0.63**
PS10	Charity Policy Officer	**0.78X**	−0.02	0.13	**0.42**	−0.02	**0.78**
PS05	Political Campaigner	**0.78X**	0.04	0.18	0.16	0.2	**0.65X**
PS07	Economic and Social Initiatives	**0.75X**	−0.08	0.15	**0.54X**	0.26	0.38
PS15	Social Policy - Finance	**0.75X**	0.02	0.40	0.18	0.18	**0.81X**
PS12	Social Care Service Coordinator	**0.73X**	0.08	0.37	0.39	0.00	**0.61X**
PS25	Doctor (Hospital Based Medicine)	**0.72X**	0.20	0.26	0.26	0.37	**0.61X**
PS11	Social Activist	**0.72X**	**−0.47**	0.28	**0.77X**	0.17	0.27
CP08	Diarist - Money Advice	**0.71X**	0.07	−0.11	0.04	**0.43**	**0.49**
CP04	Diarist - Enterprise Loan	**0.70X**	−0.13	−0.36	**0.64X**	0.32	0.26
PS14	Policy and Poverty	**0.69X**	−0.39	0.35	0.38	0.01	**0.66X**
PS18	Community Volunteer	**0.67X**	−0.24	0.14	**0.64X**	0.03	0.35
PS09	Community Development Officer	**0.66X**	−0.27	0.30	**0.81X**	0.12	0.26
CP06	Diarist - Enterprise Loan	**0.66X**	−0.33	0.24	**0.68X**	0.14	0.09
PS22	Community Development Worker	**0.64X**	−0.28	0.21	**0.49**	−0.15	**0.59**
CP10	Diarist - Enterprise Loan	**0.64X**	−0.19	0.19	**0.60X**	0.23	0.21
PS08	Financial Services Practitioner	**0.61X**	0.05	0.29	**0.47X**	0.40	0.25
CP23	Focus Group - Low-Income Community	**0.61X**	0.09	0.02	**0.55**	**0.55**	0.31
PS28	Financial Inclusion Researcher	**0.60X**	0.17	0.34	**0.58**	0.12	**0.51**
CP22	Focus Group - Low-Income Community	**0.59X**	−0.06	−0.10	**0.55X**	0.32	0.37
CP17	Diarist - Personal Loan	**0.58X**	**−0.46**	0.17	**0.54**	**0.45**	0.22
CP21	Diarist - Enterprise Loan	**0.52X**	0.17	−0.01	0.39	**0.55X**	0.22
CP11	Diarist - Control	**0.52X**	−0.03	0.20	**0.47X**	0.03	0.31
CP24	Focus Group - Low-Income Community	0.35	**0.64X**	−0.09	0.26	0.16	0.36
CP13	Diarist - Money Advice	0.29	**0.57X**	0.34	0.38	**0.58X**	−0.07
CP02	Diarist - Personal Loan	0.21	**0.51X**	**−0.6**	−0.08	**0.43X**	−0.15
CP01	Diarist - Personal Loan	**−0.44**	**0.50X**	0.06	−0.08	**0.48X**	0.13
CP19	Diarist - Enterprise Loan	−0.39	**0.49X**	0.25	0.35	**0.42X**	**−0.47**
PS27	Social Policy Researcher	0.27	−0.03	**0.60X**	**0.54X**	0.25	0.33
PS24	Social Worker	0.07	0.29	**0.59X**	**0.60X**	0.40	0.25
PS19	Community Development Officer	0.05	0.25	**0.58X**	**0.48**	**0.58**	0.19
PS23	Health Professional	0.13	0.08	**0.57X**	**0.54**	0.39	**0.45**
PS17	Community/Enterprise Development	0.07	−0.04	**0.49X**	**0.67X**	0.28	−0.01
CP03	Diarist - Personal Loan	0.28	0.00	**0.44X**	0.02	0.10	−0.08
PS02	Public Health and Social Policy Researcher	**0.67**	−0.09	**0.47**	**0.67**	−0.10	**0.51**
PS26	Academic Public Health Researcher	**0.62**	−0.03	**0.53**	0.23	0.03	**0.86X**
PS20	Academic Poverty, Health and Social Policy Researcher	**0.61**	−0.01	**0.45**	**0.68**	−0.09	**0.42**
PS03	Public Health Consultant	**0.59**	−0.08	**0.6**	**0.46**	0.37	**0.51**
PS06	Local Government Policy Officer	**0.49**	−0.08	**0.53**	**0.59**	0.00	**0.41**
CP25	Focus Group - Low-Income Community	0.26	−0.4	0.12	**0.65X**	0.30	0.12
CP18	Diarist - Money Advice	0.26	0.11	0.16	0.08	**0.51X**	0.36
CP16	Diarist - Enterprise Loan	0.11	0.27	0.23	**0.58X**	0.19	0.17
CP05	Diarist - Enterprise Loan	0.00	0.11	0.02	**0.49**	**0.46**	−0.01
CP07	Diarist - Enterprise Loan	−0.06	0.26	0.08	0.30	**0.57X**	0.06
CP20	Diarist - Enterprise Loan	−0.08	0.39	0.03	0.37	0.35	0.27
CP14	Diarist - Money Advice	−0.08	−0.25	− 0.30	0.08	**0.60X**	−0.06
CP09	Diarist - Money Advice	−0.25	−0.10	− 0.06	0.31	**0.48X**	0.20
CP12	Diarist - Control	0.29	0.42	−0.04	0.23	**0.54X**	0.37
CP15	Diarist - Control	−0.41	0.40	−0.11	**0.50**	**0.48**	0.05
**% Explained Variance**		**31.00**	**7.00**	**11.00**	**22.00**	**11.00**	**20.00**

* The table is ordered by factor loadings on ‘Causes’. Significant loadings (*p* < 0.01) are shown in bold type. The significance level is calculated as 2.58*(SE). SE represents standard error that is defined as 1/√N where N is the number of statements in the statement set. For ‘Causes’, 2.58*(SE) = 2.58 (1/√34) = 0.44. For ‘Solutions’, 2.58*(SE) = 2.58 (1/√39) = 0.41. Defining sorts are identified by an X. ‘PS’ = Professional Stakeholders. ‘CP’ = Community Participants

## Results

Fifty-three respondents (28 professional stakeholders located broadly within the central belt of Scotland and 25 community participants from in and around the city of Glasgow – see Table 3) completed a card-sort for ‘Causes’ and for ‘Solutions’. Table 4 shows three factor solutions were statistically supported and yielded interpretable accounts consistent with qualitative data. Tables 1 and 2 indicate the position of each statement in the idealised card-sorts for each factor.

All 53 card-sorts are associated to some degree, positively or negatively, with the three accounts in each factor solution (see Table 4). Using a significance level of *p* < 0.01 ‘Causes’ factors^1^ are defined (identified by an ‘X’ in Table 4) by 27, 5 and 6 card-sorts respectively, and ‘Solutions’ factors are defined by 15, 10 and 9 card-sorts (see Tables 3 and 4); for example, PS13 defines ‘Causes’ F1 (see Table 4). Card-sorts which have significant loadings on more than one factor are called ‘mixed loaders’, for example, PS22 in ‘Solutions’ (see Table 4), and card-sorts which do not load significantly on any factor are called ‘null loaders’, for example, CP05 in ‘Causes’ (see Table 4). No professional stakeholders defined accounts C-2 and S-2 and no community participants defined S-3 (see Table 3).

### Factor descriptions

Factor descriptions are a narrative based on the placement of statements in the idealised card-sort for each factor and quotes from post-sort interviews. Short factor descriptions are presented in the following sub-sections for ‘Causes’ and ‘Solutions’ (see Additional file 2 for full descriptions referencing placement of specific statements and quotes from respondents who defined each factor).

### ‘Causes’

#### C-1: ‘Unfair society’

The main causes of health are structurally determined, through the economy and employment, the welfare system and housing, and further influenced by the politicised environment in which these structures operate. Securing a job is a struggle and those that are available tend to be of low quality and transitory. This negatively impacts individuals’ sense of self-esteem, their ability to exert control over their lives and money issues. Having unpredictable finances causes daily stresses that undermine one’s ability to forward plan. The welfare system does not ease these concerns as individuals receive insufficient resources, it is too inflexible to cater for individuals’ changing circumstances and their users are stigmatised and portrayed in popular culture as being dependent and lazy. It is offensive to suggest that poor health in poor communities is explained by people not taking responsibility for their own health or being unable to look after themselves.

#### C-2: ‘Dependent, workless and lazy’

Poor health in poor communities stems from individuals’ lost ability and motivation to properly look after themselves due to their over-reliance on the state. Many low-income people have a complicated relationship with employment: some are lazy and want to rely on the welfare system, others want to work but cannot find jobs and some have jobs but these are insecure and offer no sense of purpose. Thus there is a tendency towards worklessness and dependence on the state with individuals constantly worrying about money and being unable to exert control over their lives. These factors have led to individuals being conditioned and *cleansed* of their ability to look after themselves. This alongside their lack of ambition impacts on their outlook and behaviour as there is a tendency to focus on short-term pleasures as a distraction from life; society makes them feel worthless so they behave as such.

#### C-3: ‘Intergenerational hardships’

Worse health in low-income communities comes from complicated, intergenerational family situations which have worsened because of poorly targeted government investment and policies. Poor children are more likely to suffer, or be witness to, some form of abuse – physical, sexual or emotional. Role models are lacking and children can suffer the debilitating effects of poor parenting. These early life experiences have many knock-on effects: children’s outlook on life is negatively affected; they struggle to exert control over their life; have difficulty gaining a good education and a good job when they grow up. While there has been investment in housing and physical regeneration this is often not done well and money has not been spent on economic and social infrastructure. Insecure jobs with poor pay causes stress and unpredictable finances mean individuals lack financial resilience. Individuals have nothing to be motivated for, feel excluded and have daily struggles to get through the day.

### ‘Solutions’

#### S-1: ‘Empower communities’

Health would be improved by devolving power to communities so they can decide what needs to be done. There is no point doing more of the same and expecting different results, such as running more health campaigns. Having decision making responsibility will help empower communities and develop social connectedness as people come together to make decisions. Individuals need to feel that they belong somewhere and feel like they have something to do and this is best achieved by investing in community activities and groups. Feeling connected to something can reduce isolation and loneliness and boost self-confidence and self-esteem. There is a need to help people in these communities, especially vulnerable groups like children, to develop strengths and relationships. Money is needed to cover basic needs as this will enable them to prioritise other aspects of their life and provide them with greater opportunities. Punitive policies that reduce the amount of money individuals have, such as cutting or denying welfare benefits, or access to health care should be avoided as this would only worsen a bad situation.

#### S-2: ‘Paternalism’

To improve health supportive frameworks should be put in place to enable people in low-income communities to make better choices. The creation of an environment in which it is easier to be healthier would involve the alignment of incentives and investment to nudge people into looking after themselves. To reduce welfare claims more employment opportunities need to be created. People on a low-income need protection from payday and doorstep lenders and would benefit from the provision of money advice services, which would help them gain some control over their financial lives and alleviate associated worry and anxiety. Penalising welfare recipients who spend money on drugs or alcohol could be an option. Children need to be better educated about health and eating habits and the price of healthy food should be reduced. The availability of health professionals, such as general practitioners (GPs), and mental health services needs to be increased in communities and schools.

#### S-3: ‘Redistribution’

Fundamental, structural changes are required, targeting the distribution of income, wealth and power in society. Individualised interventions or those that aim to shift responsibility to the community without giving them the necessary resources to improve health will not work. The main mechanism available to enact change is the tax system which needs to be more progressive so those who have more contribute more. This would fund well-resourced public services – childcare, social housing, cheaper public transport, primary/ social care – and help to reduce toxic inequities, which make people who are already materially worse-off feel even worse. Good work needs to be available and welfare benefits should not be cut. It is important to make sure basic needs can be met and initiatives, such as a Citizen’s Basic Income should be explored. Such policies would reduce the vulnerability of individuals and help make society more equitable.

#### Exploring the relationship between ‘Causes’ and ‘Solutions’

Table 5 shows Pearson correlations between the factor loadings of each ‘Causes’ factor and each ‘Solutions’ factor (from Table 4). C-1 has a high positive statistically significant association with S-3 (0.72) and a statistically significant negative association with S-2 (− 0.58). While the size of association is not high between C-2 and ‘Solutions’ the direction of effect is clear and the relationships are statistically significant; C-2 is positively associated with S-2 (0.40) and negatively associated with S-1 and S-3 (− 0.42 and − 0.28). ‘Causes’ factor C-3 has a positive statistically significant association with ‘Solutions’ S-1 and S-3 (0.36 and 0.29) and has a negative statistically significant association with S-2 (− 0.38).

**Table 5:** Correlations between ‘Causes’ and ‘Solutions’

		Solutions		
		S-1: Empower communities	S-2: Paternalism	S-3: Redistribution
Causes	**C-1:Unfair society**	0.20	−0.58***	0.72***
	**C-2: Dependent, workless and lazy**	−0.42***	0.40***	−0.28**
	**C-3: Intergenerational hardships**	0.36***	−0.38***	0.29**

*** 1% significance level; ** 5% significance

## Discussion

This study has shown that plural views exist among professionals and community participants relating to the perceived causes of, and solutions to, health inequalities. Relationships between ‘Causes’ and ‘Solutions’ and between and within societal groups were also explored.

### Findings between and within professional and community participant groups

Amongst professional stakeholders there were different understandings of causes of, and solutions to, health inequalities. These accounts (C-1, C-3, S-1 and S-3) are reflected in broader debates within the public health literature between salutogenesis and asset-based approaches and those who emphasise a material inequalities position [41, 42]. Notably, no professional participants defined accounts that emphasised individual responsibility and behaviour (C-2 and S-2). Community participants, on the other hand, contributed to all three ‘Causes’ factors, which reflects findings from a recent meta-ethnographic review [8]. However, even among those identifying structural causes (C-1) as the main problem, structural solutions (S-3) were not recognised. It is unclear why this is the case, but one explanation could be community participants internalising an individual responsibility discourse in the UK of ‘strivers’ and ‘skivers’ with welfare recipients being particularly stigmatised and prejudiced [43, 44]. The lack of resonance with structural solutions among community participants is similar to qualitative findings in the USA [23] and Australia [24] highlighting this is not unique to the UK.

It must be stressed that we are not making claims about the representativeness of these accounts, for example, that *no* community participants hold S-3; this is not the purpose of Q methodology and requires further work to explore the distribution of viewpoints [45]. A limitation of our study is that the professional sample is mostly comprised of public health, social policy and community development experts; it is possible that other professional stakeholders not sampled in this study could be associated with C-2 and S-2. Furthermore, a sample of community participants was not significantly associated with any factor. While no new shared views were found from a factor analysis of the community participant sample only it is possible another shared view could be revealed from recruitment of more community participants.

### ‘Causes’ to ‘Solutions’

Exploration of the relationship between ‘Causes’ and ‘Solutions’ found viewpoints align as expected given qualitative descriptions of the accounts. Beliefs that structural factors are to blame for the worse health of low-income communities meant solutions that emphasised the need to change the way income, wealth and power are distributed were expressed (C-1 and S-3). Similarly, the adoption of a more paternalistic approach was positively associated with the view that the insufficient ability/ lost motivation of individuals was responsible for their worse health (S-2 and C-2). Interestingly, the view that worse health of individuals was inevitable because of intergenerational, family issues (C-3) was positively associated, albeit with lower correlations, with two solutions (S-1 and S-3). S-1 highlights the need to empower communities and improve connectivity and fits with concerns about isolation and lack of control (C-3). Recognising the need for better jobs and the importance of meeting basic needs (S-3) aligns with a view which acknowledges that insecure working conditions and money worries can negatively affect health (C-3). While the Pearson correlations in Table 5 are based on a relatively small sample of data (*n* = 53) which could affect the relationships identified, this analysis produces statistically significant and qualitatively meaningful results.

### Consensus amongst disagreement

Despite the plurality of views revealed, there were important consensus issues, most notably relating to money. There is consensus that unpredictability of finances (#6) and job insecurity (#15) lead to worse health and that welfare benefits should not be cut as a way to improve health (#14). Moreover, the importance of making sure people have enough money for their basic needs (#13) as a way to improve health was important for all accounts. While these findings do not offer prescribed policies they do point to the importance of income security and having enough money for basic needs. Consensus in these groups points to a way forward that might find general support from experts and communities. Modelling work also illustrates the potential effectiveness of regulatory and tax options, such as a ‘living wage’, welfare benefit increases and changes to income taxes, for reducing health inequalities [46]. This raises questions for policymakers and public health research: to continue to focus more on ‘downstream’, individualised interventions that are easier to evaluate and implement or to listen to what community members with lived experience and experts are telling us, that acting ‘upstream’ on material resources is necessary for reducing health inequalities.

## Conclusion

Use of Q methodology enabled us to explore perceptions of the causes of, and solutions to, health inequalities among and between different societal groups and how beliefs about causes relate to beliefs about solutions. We found different narratives exist on this topic, which is perhaps not surprising, and the study illustrated the nature of this plurality and areas of consensus. Professional stakeholders and community participants share accounts that view structural and generational issues as the causes of poorer health in low-income communities, which are associated with upstream solutions based on redistribution and empowerment. However, community participants also focused on more-individualistic causes and paternalistic solutions that were not found among professional stakeholders. Interestingly, across all parties and perspectives there was broad agreement on the importance of money for health inequalities around, for example, issues relating to unpredictable finances, job security, not cutting welfare benefits and having enough money for basic needs. This consensus is important as it points a way forward for tackling health inequalities, highlighting areas for policy and future research.

## Additional files

Additional file 1: Context; a standardised introduction that set the context for the card-sorts. (DOCX 13 kb)

Additional file 2: Full Factor Descriptions; full descriptions for the ‘Causes’ and ‘Solutions’ factor solutions referencing placement of specific statements and quotes from respondents who defined each factor. (DOCX 24 kb)

## Abbreviation

(GPs): General practitioners

## Footnotes

1 Within our ‘Causes’ factor solution there was a sample of community participants who were not significantly associated with any factor (‘null loaders’). This could suggest another shared view exists among our community participants not identified by our analysis. However, a separate factor analysis of the community participant sample only, to explore this issue, did not reveal new shared viewpoints.

## References

[1] Marmot M (2010). Fair society: healthy lives. Strategic review of health inequalities in England Post-2010.

[2] McCartney G, Collins C, Walsh D, Batty G (2012). Why the scots die younger: synthesizing the evidence. Public Health.

[3] Whitehead M (2007). A typology of actions to tackle social inequalities in health. J Epidemiol Community Health.

[4] Wilkinson RG, Pickett KE (2009). The Spirit level: why more equal societies almost always do better.

[5] Blaxter M (1997). Whose fault is it? People's own conceptions of the reasons for health inequalities. Soc Sci Med.

[6] MRC (2008). Developing and evaluating complex interventions: new guidance. Medical Research Council.

[7] Popay J, Williams G, Thomas C, Gatrell A (1998). Theorising inequalities in health: the place of lay knowledge. Sociology of Health and Illness.

[8] Smith KE, Anderson R. Understanding lay perspectives on socioeconomic health inequalities in Britain: a meta-ethnography. Sociology of Health and Illness. 2017:1–25. 10.1111/1467-9566.12629 29044572

[9] Mackenzie M, Collins C, Doyle M, McCartney G (2017). Working-class discourses of politics, policy and health: ‘I don’t smoke; I don’t drink. The only thing wrong with me is my health’. Policy Polit.

[10] Garthwaite K, Bambra C (2017). “How the other half live”: lay perspectives on health inequalities in an age of austerity. Soc Sci Med.

[11] Douglas M, Smith KE, Bambra C, Hill SE (2016). Beyond ‘health’: why don’t we tackle the cause of health inequalities?. Health inequalities.

[12] Katikireddi SV, Higgins M, Smith K, Williams G. Health Inequalities: The Need to Move Beyond Bad Behaviours. Journal of Epidemiology and Community Health. 2013. 10.1136/jech-2012-202064 23486925

[13] Graham H (2009). Health inequalities, social determinants and public health policy. Policy Polit.

[14] Bambra C, Gibson M, Sowden A, Wright K, Whitehead M, Petticrew M (2010). Tackling the wider social determinants of health and health inequalities: evidence from systematic reviews. J Epidemiol Community Health.

[15] Ogilvie D, Egan M, Hamilton V, Petticrew M (2005). Systematic reviews of health effects of social interventions: 2. Best available evidence: how low should you go?. J Epidemiol Community Health.

[16] Petticrew M, Whitehead M, Macintyre SJ, Graham H, Egan M (2004). Evidence for public health policy on inequalities: 1: the reality according to policymakers. J Epidemiol Community Health.

[17] Barr B, Bambra C, Smith K, Smith KE, Bambra C, Hill SE (2016). For the good of the cause: generating evidence to inform social policies that reduce health inequalities. Health inequalities.

[18] Mackenbach JP (2011). Can we reduce health inequalities? An analysis of the English strategy (1997—2010). J Epidemiol Community Health.

[19] Masters R, Anwar E, Collins B, Cookson R, Capewell S. Return on investment of public health interventions: a systematic review. J Epidemiol Community Health. 2017:1–8. 10.1136/jech-2016-208141 PMC5537512 28356325

[20] Ndumbe-Eyoh S, Moffatt H (2013). Intersectoral action for health equity: a rapid systematic review. BMC Public Health.

[21] Smith K (2013). Beyond evidence-based policy in public health: the interplay of ideas: Palgrave Macmillan.

[22] Haslam SA, McMahon C, Cruwys T, Haslam C, Jetten J, Steffens NK (2018). Social cure, what social cure? The propensity to underestimate the importance of social factors for health. Soc Sci Med.

[23] Lundell H, Niederdeppe J, Clarke C (2013). Public views about health causation, attributions of responsibility, and inequality. J Health Commun.

[24] Putland C, Baum FE, Ziersch AM (2011). From causes to solutions - insights from lay knowledge about health inequalities. BMC Public Health.

[25] Mackenzie M, Hastings A, Babbel B, Simpson S, Watt G (2017). Tackling and mitigating health inequalities – policymakers and practitioners ‘talk and draw’ their theories. Social Policy and Administration.

[26] Smith KE, Eltanani MK (2014). What kinds of policies to reduce health inequalities in the UK do researchers support?. J Public Health.

[27] Ritchie J, Lewis J, McNaughon Nicholls C, Ormston R (2014). Qualitativ research practice: a guide for social science students & researchers. National Centre for social research: SAGE publications.

[28] Stephenson W (1953). The study of behavior: Q-technique and its methodology.

[29] Watts S, Stenner P (2012). Doing Q methodological research – theory method and interpretation.

[30] McKeown B, Thomas D (2013). Q methodology.

[31] Brown SR. Political subjectivity: applications of Q methodology in political science. London: Yale University Press; 1980 1980///.

[32] Mattson DJ, Clark SG, Byrd KL, Brown SR, Robinson B (2011). Leaders’ perspectives in the Yellowstone to Yukon conservation initiative. Political Science.

[33] Mattson DJ, Byrd KL, Rutherford MB, Brown SR, Clark TW (2006). Finding common ground in large carnivore conservation: mapping contending perspectives. Environ Sci Pol.

[34] Chamberlain EC, Rutherford MB, Gibeau ML (2011). Human perspectives and conservation of grizzly bears in Banff National Park. Canada Conservation Biology.

[35] Jeffares S (2014). Interpreting hashtag politics: policy ideas in an era of social media: Palgrave macmillan UK.

[36] Dahlgren G, Whitehead M (1991). Policies and strategies to promote social equity in health.

[37] Baker R, Thompson C, Mannion R (2006). Q methodology in health economics. J Health Serv Res and Policy.

[38] Schmolck P, Atkinson J (2002). PQMethod software and manual.

[39] Brown SR (1980). Political subjectivity.

[40] Kline P (1994). An easy guide to factor analysis.

[41] Morgan A, Ziglio E (2007). Revitalising the evidence base for public health: an assets model. Promot Educ.

[42] Friedli L (2012). ‘What we’ve tried, hasn’t worked’: the politics of assets based public health. Crit Public Health.

[43] Baumberg B (2016). The stigma of claiming benefits: a quantitative study. Journal of Social Policy.

[44] Valentine G, Harris C (2014). Strivers vs skivers: class prejudice and the demonisation of dependency in everyday life. Geoforum.

[45] Mason H, Collins M, McHugh N, Godwin J, Van Exel J, Donaldson C (2018). Is “end of life” a special case? Connecting Q with survey methods to measure societal support for views on the value of life-extending treatments. Health Econ.

[46] McAuley A, Denny C, Taulbut M, Mitchell R, Fischbacher C, Graham B, et al. Informing investment to reduce inequalities: a modelling approach. PLoS One. 2016:1–20. 10.1371/journal.pone.0159256 PMC4972318 27486857

