# Supplementary material for: Who knows best? A Q methodology study to explore perspectives of professional stakeholders and community participants on health in low-income communities
Source: BMC Health Serv Res. 2019 Jan 14;19:35. doi: 10.1186/s12913-019-3884-9 (PMC6332861; doi:10.1186/s12913-019-3884-9)
Supplement: Supplementary file 1 — Context; a standardised introduction that set the context for the card-sorts. (DOCX 13 kb) [file 12913_2019_3884_MOESM1_ESM.docx]

**Additional File 1 – Context**

In the UK, there are large differences in health between groups in society. Differences in health can be caused by many different reasons and there are a number of different things that could be done to improve health.

In this project, our focus is on the health of low-income communities. We are considering low-income communities that are similar to ones that might be found in and around major cities in the UK, such as Glasgow.

We are interested in why you think health is worse in low-income communities and the ways you think health could be improved in these same communities. As you’ll see we are not researching specific diseases but rather the causes of poor health more generally.

Do you have any questions so far?

Would you like me to go over any part of this explanation again?
