# Supplementary material for: Who knows best? A Q methodology study to explore perspectives of professional stakeholders and community participants on health in low-income communities
Source: BMC Health Serv Res. 2019 Jan 14;19:35. doi: 10.1186/s12913-019-3884-9 (PMC6332861; doi:10.1186/s12913-019-3884-9)
Supplement: Supplementary file 2 — Full Factor Descriptions; full descriptions for the ‘Causes’ and ‘Solutions’ factor solutions referencing placement of specific statements and quotes from respondents who defined each factor. (DOCX 24 kb) [file 12913_2019_3884_MOESM2_ESM.docx]

# **Additional File 2 – Full Factor Descriptions**

Notation is used in the following descriptions: # indicates statement number, and is followed by the position of that statement on the grid for that factor. For example, (#15, +3) indicates that statement number 15 appeared in position +3 for the factor in question.

# **‘Causes’: Health is worse in low-income communities because . . .**

# **C-1: ‘Unfair society’**

# The main causes of health are structurally determined, through the economy and employment, the welfare system and housing, and further influenced by the politicised environment in which these structures operate. The nature of the economy means that individuals struggle to secure jobs and those jobs that do become available tend not to offer meaningful employment because they are of low quality and a transitory nature (#15, +3; #32, +2). As well as negatively impacting on individuals’ sense of self-esteem and the control they exert over their lives, it leads to money issues (#16, +1). Unpredictable finances cause daily stresses of having to make difficult decisions in order to get by (#9, +4; #6, +3) which not only impacts on mental health issues but *“hampers your ability to look forward and to plan for anything beyond a day”* (PS16). This further aggravates the “*poverty premium*” (PS16) low-income individuals face where services and utilities cost more because they have less money (#26, +3).

# Ironically, the welfare system which should be set-up to ease these concerns is “*so bad that it must be deliberately intended not to work well for those people*” (PS04). Individuals receive insufficient resources and it is too inflexible to cater for individuals’ changing circumstances (#13, +4; #14, +2). Moreover, political parties and media are responsible for perpetuating a stigmatising narrative around recipients being dependent and lazy (#30, -4; #13, +4; #20, +2) to create acceptance of social, economic and health inequalities in order to “*prevent … social change*” (PS01). This leads to low-income individuals being stereotyped and feeling excluded which contributes to their already low sense of self-worth from living in poor conditions due to lack of government investment in social housing (#32, +2; #11, +2; #20, +2; #3, +1). Instead of recognising that “*the least lazy people in society are those that have to scrape and scrimp and feed their kids and make their way with such low incomes. It’s hard work being homeless. It’s hard work being on benefits. It’s hard work living in a low income community*” (PS01), the media label “*low income families as being feckless, out of control in terms of not taking control of birth control, or expecting the state to look after their children when they are unable to”* (PS16). Consequently, this leads to accusations that individuals are fully to blame for their own poor health.

# These accusations are rejected and it is offensive to suggest that people in these communities don’t take responsibility for their own health or aren’t able to look after themselves (#33, -4; #31, -3; #17, -3; #28, -2; #34, -2). Individual behaviours and the culture of the community are not to blame (#27, -3; #34, -2; #4, -2; #19, -2) rather the circumstances of their lives and the unavailability of support mean they have a reduced capacity to cope with the difficulties they face (31, -3; #17, -3; #14, +2).

**C-2: ‘Dependent, workless and lazy’**

Poor health in poor communities stems from individuals lost ability and motivation to properly look after themselves due to their over-reliance on the state. Many low-income people have a complicated relationship with employment: some are thought to be lazy and want to rely on the welfare system, others want to work but can’t find jobs and some have jobs but these are insecure and offer no sense of purpose (#30, +4; #15, +3). Thus there is a tendency towards worklessness, which is the norm in low-income families, and is a major factor in their dependency on the state (#30, +4). Families’ inability to exert control over their lives over multiple generations through access to secure and meaningful employment (#16, +1; #15, +3) has meant individuals in these communities have become “*conditioned*” (CP19) which has resulted in a “*formal cleansing of people’s innate ability to be able to look after themselves”*. Consequently, they don’t follow health advice (#28, +2) nor do they think about the future (34, +3). For example, despite having turbulent financial lives in the form of low income (#9, +4; #26, +2) and unpredictable finances (#6, +2) they will spend any money they do have as soon as they receive it on short term pleasures, such as takeaways, the cinema or holidays (#34, +3) as “*a way to distract them from life*” (CP19). This occurs despite individuals’ suffering from depression and anxiety and other mental health issues from their struggle to cope with unexpected events or costs (#23, +3) and being “*worried about money all the time*” (CP13).

Health isn’t worse because low-income communities lack services, socialising space and activities (#14, -4; #1, -4; #2, -3) these are available and of good quality. Instead the culture of the society and of these low-income communities impacts on individuals’ outlook on life and correspondingly how they act, as individuals lack ambition and society looks down at them (#19, +1, #20, +1):

“*they feel that the rest of the society don’t see them like a person or human beings, or this kind of stuff, and they feel themselves worthless. And if someone is starting to feel, or you make someone feel they are worthless, eventually they will believe that themselves. You know what I mean? And if somebody thinks he is worthless he will behave as though he is worthless. They don’t care anymore about things*” (CP01).

**C-3: ‘Intergenerational hardships’**

Worse health in low-income communities comes from complicated, generational family situations which have only got worse because of poorly targeted government investment and policies. Children are more likely to grow up in families where they suffer some form of extreme abuse – emotional, sexual or physical – or live in an environment where alcohol or drug abuse goes on in the family which causes them to have a horrendous outlook on life (#18, +4; #27, +2; #29, +1). The cycle of intergenerational family issues “*where it goes from say grandparent to parent to child*” (PS19) is extremely hard to break without the necessary support and means children really struggle to exert any control over their life (#16, +4) and can feel that if they open up about their experiences they’ll be excluded from their community (#3, +2). Instead of having good role models (#27, +2; #18, +4; #19, +1) children can grow up in a family environment where they suffer the debilitating effects of parents “*constantly telling a young person that they’re nothing or they’re this or that, that has a long term effect on your emotional health which then impacts on your physical health because you don’t look after yourself”* (PS24). The knock-on effects of this mean it’s harder to gain a good education which affects your ability to get a job and if you feel really low you will “*not be of the mind set”* (PS19) to access available space or places to meet others (#5, +3; #15, +3; #1, -3; #25, -3; #10, -2).

While government have invested in these communities, particularly in housing and physical regeneration, often it is not done well (#32, -3; #11, +3). Also government spending “*has probably not been in the right places and in the right way*” (PS27) with investment needed in economic and social, as well as physical, areas to help break intergenerational issues and community cultures (#5, +3; #15, +3; #32, -3; #19, +1). For example, those who get a job struggle to get a decent job which is important as if “*it's a rubbish job with very poor pay, it actually makes you more stressed and have an even worse impact on your health*” (PS27) (#16, +4; #15, +3; #26, +1). This can lead to unpredictable finances which can cause individuals to have a lack of “*financial resilience*” (PS27) whereby they find it difficult to cope with unexpected events and costs (#6, +2; #23, +1). People in low-income communities aren’t lazy instead “*they’ve got nothing to get up for”* (PS24) and so feel excluded from society; supporting them to access work is one way to help integrate them in society (#30, -4; #15, +3; #3, +2). It’s the multitude of issues which low-income families have had to deal with on a daily basis over subsequent generations rather than some lazy stereotype about them having too many children (#33, -4) that is the cause of their worse health as they are just *“thinking about getting through the day****”*** (PS24).

**‘Solutions’: Health could be improved in low-income communities by . . . . . .**

**S-1: ‘Empower communities’**

A humane and caring society is one where people try to help others improve their health (#34, -4). This would best be done by devolving power to communities rather than doing more of the same and expecting different results, such as running more health campaigns (#5, +5; #25, -3).

Individuals need to feel that they “*belong somewhere*” (PS17) and feel like they have “*something to do*” (PS17) and this is best achieved by investing in community activities and groups (#5, +5). This way individuals can participate in what is happening locally and so feel “*connected to what is going on*” (PS09). Importantly though, it must be the community which decides and takes responsibility for what happens within it rather than decisions being made for them (#21, +5; #38, +4; #36, -4; #16, -3). Even if mistakes are made this autonomy will help individuals and community regain control over their lives. Moreover, this empowerment of the community and the development of individuals’ social connectedness within it can help to reduce one’s sense of isolation and loneliness and boost self-confidence and self-esteem (#9, +4; #8, +4; #19, +2) – community space and groups can enable individuals to “*feel they can be the best that they are*” (PS17). Everyone within the community should be supported and helped to develop the confidence, skills and knowledge that they need for life especially vulnerable groups like children and ex-offenders (#8, +4; #24, +3; #6, +3; #20, +3; #3, +2).

Individuals in low-income communities also need enough money to cover their basic needs as this will then allow them to prioritise other aspects of their life and provide them with greater opportunities (#13, +2; #1, +2). In no way should welfare benefits be cut or denied to people for any reason (#14, -4; #33, -5); these are “*a lifeline*” (PS09) for most people. Penalising individuals by reducing their access to them would only marginalize them further and cutting benefits would take *“people down to below subsistence existence”* (PS11). Similarly, punitive policies that might in any way restrict individuals’ access to health care are rejected (#32, -5); access to health care is seen “*as a basic human right”* (PS09) and its denial would only serve to further discriminate against certain individuals.

**S-2: ‘Paternalism’**

To improve health supportive frameworks should be put in place to enable people in low-income communities to make better choices (#38, -3). This would involve the creation of an environment in which being heathier is made easier by, for example, improving individuals living conditions (#12, +2) and by introducing specific health related policies. Drug use should not be made legal (#16, -5) and an array of health related services should be augmented or introduced. For example, more GPs are needed so that it is easier and quicker to make appointments and mental health services, such as counsellors, should be provided to vulnerable people in these communities and schools (#19, +4; #37, +3; #2, +2; #6, +2). Importantly, even if individuals make poor choices they should not be denied health care (#32, -4). Rather than increasing the taxes people in these communities pay, these services could be provided from government better prioritising existing resources (#18, -4).

Another component of this framework is education. Better educating children will give them the best chance in life, particularly in relation to health (#20, +5; #24, +3) because if you pick-up “*good habits early you will carry them through with you for the rest of your life*” (CP12). Poor eating habits among adults is seen as stemming from a lack of knowledge and awareness about what is good for you. However, a lot of people don’t have enough money to buy healthy food because of its expense and low cost, unhealthy food is more convenient (#13, +5; #14, -5; #30, +3). Reducing the price of healthy food rather than increasing or limiting the availability of unhealthy things is the best way to encourage better eating habits (#30, +3; #11, -2; #36, -4).

Individuals in these communities also have daily money issues which cause stresses, worry and anxiety as “*you’ve constantly got this on the back of your mind”* (CP12) and means it is difficult to focus on any form of self-improvement (#13, +5). While cutting benefits is a potential “*disaster*” (CP21) for people who rely on them to survive, one approach could be to penalise welfare recipients who only spend money on drugs and drink (#14, -5; #33, +1). However, for people to reduce their claims on the welfare system more employment opportunities need to be created and individuals’ skills improved (#4, +2). While loans could be helpful to people in emergencies, for example when their cooker or washing machine breaks down, getting into debt is risky because of the potential for high interest payments and vulnerable people need protecting from payday or doorstep lenders (#39, +4; #10, -3). However, money advice services *“would help a lot of people’s lives, to know where their money is going, what they are spending it on, because a lot of people just waste it”* (CP14) and in doing so would help individuals gain some control over their financial lives and alleviate some of the associated worry and anxiety (#22, +4).

**S-3: ‘Redistribution’**

Instead of tinkering around the edges with various benign downstream health interventions (#25, -3) to really improve the health of low-income communities what is needed are “*fundamental structural changes and the way in which society is organized”* (PS26). Thus the goal is to target the fundamental causes of health inequalities which are how *“we distribute income, wealth and power”* (PS01).

Having good health is not the responsibility of the individual alone (#26, -4); health is a product of the context and society in which individuals live and work and the options which are available to them (#34, -5; #32, -4; #30, +2; #11, +2). Individualised interventions (#31, -3; #8, -2) or those that aim to shift the responsibility to the community (#38, -2; #27, -2; #20, -2) without giving them the necessary resources will not improve health. The main mechanism by which inequalities in society could be reduced is through the introduction of a more progressive tax system where “*people who earn more, pay more tax”* (PS26) (#18, +5). This would serve two main functions. The first being that it would enable the funding of well-resourced public services, such as the provision of free and accessible childcare (#1, +3), building of good quality social housing (#12, +4), cheaper public transport (#28, +1) and primary and social care services which are tailored to the needs of communities (#7, +3; #37, +3; #2, +2). Secondly, the redistributive nature of this change would help to reduce the “*toxic*” (PS26) levels of inequity that exist in society which can “*make the worse-off feel even worse than they might otherwise be in that level of poverty*” (PS04) and enable people to have better opportunities to access education and jobs (#15, +4; #4, +4; #17, +1) – “*the common aspects of society*” (PS26). Importantly, there should be changes in the employment sector so that jobs offer individuals ‘good work’ in terms of liveable wages, stability and security.

A further key part of reducing societal inequalities and inequities is helping individuals to meet their basic needs (#13, +5). This is an essential starting point for improving health as “*you can’t do anything else unless those (reference to basic needs) are met”* (PS13). Meeting basic needs would also help to “*alleviate so much of the mental anxiety and worries*” and enable people “to *participate fully in society*” (PS15). One initiative that could act in this way and that should be explored is a universal “*citizen’s basic income*” (PS13). This contrasts with policy measures aimed at cutting welfare benefits which would only result in negative impacts on health and wellbeing and the decrease of vulnerable individuals’ sense of self-worth (#14, -5; #33, -4).
